# Time in Care for Older People Living in Nursing Homes

**DOI:** 10.1155/2010/148435

**Published:** 2010-06-29

**Authors:** K. B. E. Thorsell, B. M. Nordström, L. Fagerström, B. V. Sivberg

**Affiliations:** ^1^Section of Nursing, Department of Health Sciences, Faculty of Medicine, Lund University, Box 157, Baravägen 3, 221 00 Lund, Sweden; ^2^Section of Elderly, Municipality of Hässleholm, Löjtnant Granlundsväg 14, 28152 Hässleholm, Sweden; ^3^Department of Health Sciences, Buskerud University College, Pb 850, Papirbredden, Grønland 58, 3007 Drammen, Norway; ^4^University of Skövde, Pb 408, Högskolevägen 1, 54128 Skövde, Sweden

## Abstract

In order to measure actual care needs in relation to resources required to fulfill these needs, an instrument (Time in Care) with which to evaluate care needs and determine the time needed for various care activities has been developed with the aim of assessing nursing intensity in municipal care for older people. Interreliability (ICC = 0.854) of time measurements (*n* = 10'546) of 32 nursing activities in relation to evaluated care levels in two nursing homes (staff *n* = 81) has been determined. Nursing intensity for both periods at the two nursing homes comprised on average a direct care time of 75 (45%) and 101 (42%) minutes, respectively. Work time was measured according to actual schedule (462 hours per nursing home during two weeks). Given that the need for care was high, one must further investigate if the quality of care the recipients received was sufficiently addressed.

## 1. Introduction

Aging not only entails a decrease in an individual's ability to engage in activities but also entails change in the form of increased dependence on others in relation to basic life functions. It is important to investigate care needs in order to ensure that, despite decreased functional ability and activity, it is possible for an older individual living in institutions to continue living a quality life. In societies faced with a continually growing older population, in order to guarantee high quality and certainty in the content of care in an effective manner, the allocation of available resources becomes ever more important. An important condition for providing quality care is the ability to clarify and, above all else, expose the content of care work so that each individual care recipient's care needs are placed in relation to the personnel resources required. Research in Europe, Asia, and the USA has shown that the time and resources needed to fulfill individual care recipients' care needs are important factors to take into consideration [1–6]. Several researchers have studied the construction of various measurement systems designed to measure care needs [7–13]. In many ways, care work is a complex issue to describe. One possible way to describe and define care work is to measure its intensity [14, 15]. Nursing intensity can be classified as both direct and indirect care. In order to assess time allocation related to nursing intensity, some type of measurement is needed. Time studies are the most common method used [15]. By measuring the time needed for various activities, the evaluation of direct, indirect, and workplace-related time can reveal how time is allocated in relation to the care needs. Such an endeavor is often directed at maximizing patient-related time and minimizing indirect and workplace-related time. 

In order to be able to measure actual care needs in relation to resources required, an instrument to evaluate the need for care in municipal care for older people has been developed. This instrument consists of Time in Care for need (TiC-n) and Time in Care for time (TiC-t), which together compromise a patient classification system designed to describe nursing intensity and the requested time for nursing in relation to patients' care needs. The purpose of this study is to, through time studies, determine the requested time for nursing of care recipients on different care need levels (nursing intensity measured by TiC-n) and to assess the reliability and validity of TiC-t, the method for time studies.

## 2. Nursing Intensity

According to Kirkevold [16], nursing theories can be described in four dimensions: area of the nurses' responsibility, goals, methods, and context. The purpose of such classification is to chart variations in how various nursing theories describe care personnel's work tasks and their approach to said work tasks, what the goals of care work are, and in what context the work occurs. When care work is exposed emanating from the description of its intensity, which comprises its context, extent, and volume, the question of how care needs can be fulfilled is answered. 

Nursing intensity can even be related to concepts such as acuity, care intensity, nurse dependency, patient dependency, and nursing care intensity. Frilund and Fagerstrom [17] describe nursing workload and nursing intensity as closely related concepts. These concepts can be used synonymously or separately to describe direct and indirect care. As regards the concept nursing workload, no exact definition of the concept was found in the literature studied, but Rauhala [15] refers to Morris [18] who suggests that nursing workload should represent the content of nursing intensity.

## 3. Patient Classification Systems

Patient classification groups patients according to accepted diagnoses, nursing intensity, treatment, diagnosis related groups (DRG), or demographic factors [15]. Giovannetti [19] defines patient classification as the grouping of patients in accordance with an evaluation of their care needs over a certain period of time. De Graot [20] defines patient classification systems as methods and processes which estimate and prescribe the individual care needs of a patient over time in order to facilitate decisions regarding how personnel, budgetary planning, the defense of costs for care service to patients, and the measurement of quality standards can be realized. Patient classification systems are foremost and often only used to measure and compare care between wards/units and hospitals so that a more objective indication of which resources are needed and where the cost for these resources can be attributed [21]. 

The development of measuring instruments that classify care needs has resulted in two main types or models of classification systems: prototype systems and factor systems. Fagerström [14] describes these classification systems. The prototype model emanates from a relatively broad/general description of the characteristic traits of a typical patient (the prototype) for each category in the system. The estimation of a patient's care needs is summarized and compared to the prototype patient's care needs. The determination of needs using a factor system emanates from specific factors or indicators that describe patients' care needs and how much time is spent on care work's various moments. The addition of the total points (score) awarded results in various care weight classes. Several researchers describe the manner in which prototype and factor systems are formed [5, 19, 22–27]. The fundamental idea behind these various systems is that there are many different aspects which influence the time it takes to fulfill a patient's care needs: the patient's state of health, personnel's care philosophy, and proficiency level, as well as the organization of the care and the design of the physical premises where the care takes place [28, 29]. The instrument that is developed for and used in this study is based on a factor system.

## 4. Ethical Considerations

The care recipients, their relatives, and the nursing home staff were verbally informed about the study, and efforts were made to prevent any apprehension regarding observations made during the course of the study; informed consent for participation in the study was thereafter duly obtained. This study has been conducted as a quality assurance study, and the various unit heads have been informed of the purpose of the study and that the study results will be used for the purposes of research. In that one of the study researchers is employed in a medical capacity at one of the facilities participating in the study, it has been made clear that her role during the course of this study is that of researcher. Accordingly, this researcher has not participated in the measurements taken for this study but has instead merely analyzed them after completion of the measurement period. The Regional Ethics Committee, Faculty of Medicine at Lund University (LU-321-03), deemed that no further formal inquiry was needed given that the study could be viewed as routine quality assurance. Furthermore, this study fully complies with the Declaration of Helsinki, which states that all information must be conveyed to the care recipients participating in a study.

## 5. Method

### 5.1. Settings

The collection of data occurred during the spring of 2009 at two separate municipal nursing homes with a collective total of 159 care recipients during two separate weeklong periods. Both nursing homes are built in an old-fashioned way with long corridors and the same environmental design. The nursing homes have between 10–18 care recipients per ward with the average age of the care recipients being 89 years and 86% being female. Five nursing assistants worked every day from 6.45 AM until 9.15 PM in each ward; staff's combined work hours equated to 33 hours per day. At night, 4–6 nursing assistants were responsible for the entire institution at both of the homes, but these carers are not included in this study. Registered nurses (RN) were on hand on a consultative basis from 8 AM until 5 PM Outside of these hours, RNs were available (on-call) in case of emergency. However, these RNs are not included in this study due to the fact that the continuity of the measurements could not be assured. Substitute carers not normally employed by the nursing homes were working at the institutions at the time. The collection of data occurred at the two nursing homes during the day with a one-week interval between the two measurements. The period measured each day and per ward was 13 hours and 30 minutes. The total amount of hours, when the time studies were conducted, was 462 hours per nursing home during two weeks), in all 924 hours.

### 5.2. Instruments and Data Collection

The TiC-n (2008) instrument classifies the care needs of care recipients residing in municipal nursing homes. The TiC-n encompasses the evaluation of individual care needs and consists of 19 items divided into three areas of need: Common care (9 items), Medical care (5 items), and Cognitive care (5 items). Each item is assessed in accordance with a numerical scale (0–4 points) where the number of points (score) describes actual care needs evaluated using a TiC-based manual. Next, the scores from these three areas of need are added together and the care recipients are thereafter classified into one of 5 care levels [13].

The TiC-t describes the amount of time used to fulfill care needs. By the TiC-t instrument, the time spent on each care activity is measured. There are three types of activities, which are activities related to direct time, indirect time, and workplace-related time. Direct time is the time spent with individual care recipients (face to face), indirect time is the time that can be related to individual care recipients but which is not spent with them, and workplace-related time is the time spent doing general work (not related to care recipients). 

In order to create a representative collection of activities to be included in the TiC-t and which most often occur in daily care work, a work group consisting of representatives from applicable personnel categories and the relevant nursing homes' heads charted the activities that occur in everyday work. These activities were thereafter tested during a pilot study which encompassed 113 care recipients [30] before a final study of all of the 505 care recipients at 13 different municipal care homes was undertaken [31]. The study ended up with 32 care activities. A questionnaire was developed including these 32 care activities and includes the evaluation of direct, indirect, and workplace-related time.

**Figure 1:**
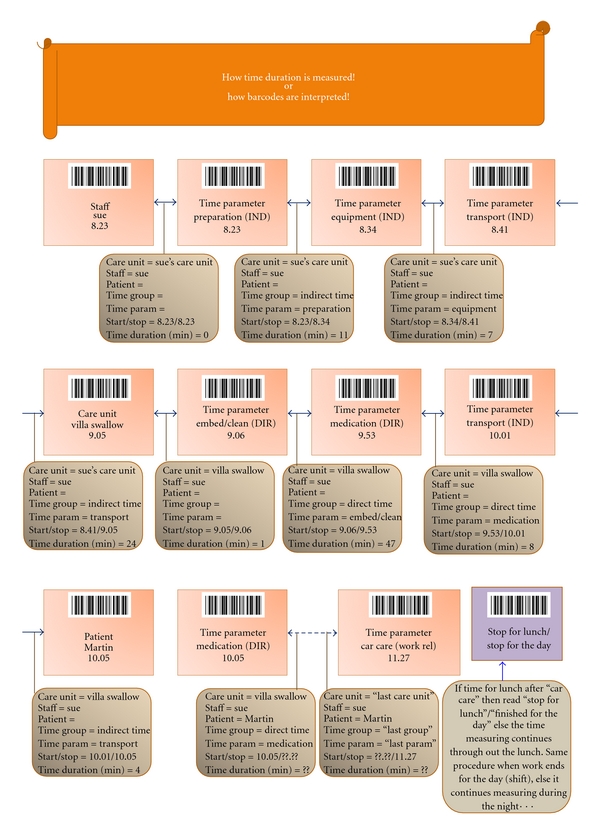
Interpretation of entered barcodes.

In this study, the care personnel registered the 32 care activities included in the questionnaire, using a barcode and a barcode scanner. The barcode scanners have a built-in clock which records the time that the activities are registered. When all care personnel, working during the study hours, registered how their time was spent, the total amount of time for meeting the recipients care needs could be calculated. An example of the flow chart of how activities are measured is seen in Figure 1. Accordingly, results pertaining to the time spent on all activities for each recipient can be calculated in direct care time and per ward and in indirect and workplace-related time. Prior to commencement of the study, all pertinent personnel were given information regarding the study's purpose and goal. During a trial week, the personnel were given the chance to test the barcode scanners. Questions pertaining to the method were thereafter discussed. The questions mainly pertained to the technique of registering activities with the barcode scanner. The questions were duly answered and explanations given, allowing measurement to begin.

### 5.3. Analysis 

A database was constructed in an Open Access environment, and each day measurements were transferred to an Excel worksheet that was imported into the database. Thereafter the data was analyzed using the SPSS (17.0) and Microsoft Excel Analyse-It software programs. In order to describe the content of the various care activities, descriptive statistics have been used. In order to investigate reliability, a test-retest was performed for both of the measurement periods, and the Inter Correlation Coefficient (ICC) was calculated at 95% confidence interval in order to explore the unanimity between both weeks' results regarding the time spent on various activities. Validation of the determined time and needs intervals was calculated using the Kruskal-Wallis test.

## 6. Results

**Figure 2:**
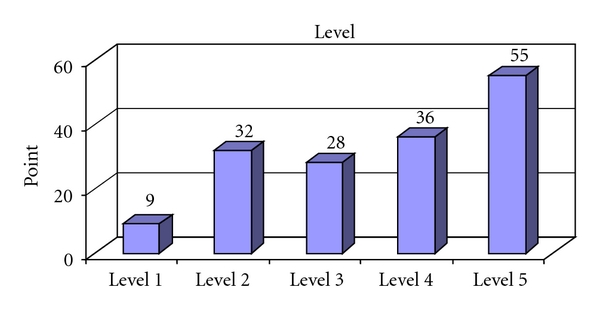
Care levels evaluated using TiC-n.

The distribution of the various care activities between the two nursing homes and two periods is described in Tables 1(a), 1(b), and 1(c) and 2(a), 2(b), and 2(c).  Figure 2 shows care recipients' individual care needs as evaluated using TiC-n. The study results show that the time spent on the care activities registered as pertaining to the fulfillment of care needs was quite low despite the fact that the care recipients' care levels were high. The intercorrelation test was completed and ICC calculated at 0.854 (*F* test,*P* = .001).

Nursing intensity between 6.45 AM and 9.15 PM for both periods one and two at the first nursing home comprised on average a direct care time of 75 minutes (45%), an indirect care time of 66 minutes (39%), and a workplace-related care time of 27 minutes (16%). The equivalent measurement at the second nursing home comprised on average a direct care time of 101 minutes (42%), an indirect care time of 79 minutes (31%), and a workplace-related care time of 66 minutes (27%).  Table 3 shows the time in minutes for each home. An average number of measurements for both periods were in nursing home one 6′237 and in nursing home two 4'309. The total frequency of each measurement and mean in minutes are shown in Table 1 and 2 (a–c). 

Regarding to the time spent on the five care levels it was obvious that in this study the greatest difference in how time was spent exists between care levels four and five.  Table 4 shows how the various care levels in relation to time spent were distributed. From the results (Kruskal-Wallis test), one sees that as the needs level increased, the mean time for all activities increased, which is statistically significant. Consequently, this result supports the validity of the TiC-t.

## 7. Discussion

The evaluation of each care recipient's individual care needs is an important condition for insuring that each individual in need of care receives the quality of care that is stipulated in Swedish legislation. If security/safety and quality are to be attained, care needs and care activities must be related to one another. By measuring both care needs and the time needed for each care activity, the content of care can be described using quantitative terms. 

Kirkevold maintains that care does not merely consist of separate activities but that care activities must instead be weighed together to form an entity in order to reflect daily care. In this study, this has occurred by calculating a total score for each care recipient which reflects his/her care needs. In addition to the evaluation of individual care needs and the time needed for such, further components must be clarified in relation to nursing intensity. Components such as proficiency and competency, area of responsibility, and care environment must be described [16].

Furthermore, the goal of care personnel's work must be taken into account [17, 32, 33]. On several occasions, traditional time studies have been used as a method for assessing how much time, and personnel are required and to describe nursing intensity. Several systems for measuring time allocation and for the classification of care needs have been strongly called into question [17, 32, 33] since critics have maintained that that which is measured merely becomes a technical and mathematical calculation that does not take into account the complexity of care. Time studies often measure one care activity at a time and many researchers [8, 14, 15, 34–38] refer to this as an obvious shortcoming. De Groot [39] maintains that care is dynamic in character and that care personnel, during their care for individual care recipients, continuously prioritize their care based on a holistic view. Researchers [40, 41] have even criticized the methods for not taking into account the quality aspects of care work and for allowing organizational leaders to use calculations as a mere economic steering instrument [14, 41, 42]. The study results showed that the need for care was relatively comprehensive measured by TiC-n and should require significant care actions. The time spent on each activity showed that, in general, the same action took the same amount of time to complete at both of the studied nursing homes. The explanation for such can lie in that care work tends to follow ingrained/practiced routines where the daily schedule determines care needs rather than the care needs that the recipients themselves have. In an overview of a paper on the care of older people in the Nordic countries [43], one finds a description of the shape that daily life for older people takes at nursing homes [44]. In Sweden, Franssén and Melin Emilsson [45, 46] have studied everyday life at nursing homes from both the personnel and care recipients' perspectives. In their studies, more focus is placed on the experiences that personnel have of their work with older people than on care recipients' perspectives. The personnel spoke of the importance of creating close relationships with care recipients and fulfilling care recipients' collective needs yet during their actual care work personnel spent most of their time meeting care recipients' physical needs and on social interaction with their work colleagues. The results also showed that physical care dominated the content of personnel's work and that, while care personnel demonstrated great personal engagement in their meetings with care recipients, they spent very little time with them.

The study itself demonstrated a good inter correlation reliability (ICC 0,854) in the sense of that the measured time for care activities to supply care needs in the two homes was to 85% unanimous. Measurements were performed with the barcode technique to ensure objectivity as far as possible as the measurements were not simultaneously carried out. The question of whether the personnel involved in this study have been influenced in any manner by the fact that one of the study researchers is employed at one of the nursing homes has been actualized. Any evidence of such influence would have been reflected in a shorter or longer period of time spent on the actual care activities at the nursing home the researcher is employed at. However, the similarity of the results from both of the nursing homes seems to dismiss the possibility of any undue influence by this researcher. The size of the study can be questioned. When comparing the inter correlation reliability, it is important that the conditions in the two nursing homes be as similar as possible. This has been taken into consideration in this study. The number of care recipients at both homes has been the same, the physical layout of the homes has been similar, and the number of staff identical. 

Examination of the time spent on various care levels has been calculated using the Kruskal-Wallis test. The results supported the study's validity in that a lower care level significantly has shown a lower time rate whereas a higher care level has shown a significantly higher time rate. The choice of study participants and their number can also be questioned. Are the two nursing homes chosen for inclusion in this study representative of all the municipality's nursing homes? The two nursing homes chosen are comparable as pertains to the number of care recipients, care recipients' age structure and sex distribution, and patient diagnoses. In order to ensure that the time spent on care activities reflects the reality that exists in other nursing homes, further data collection is needed in order to generalize the results seen here. 

In this study's results, one sees that at the two included nursing homes during both the time periods measured only one full hour per day were allotted to the fulfillment of individual care recipients' most fundamental care needs. The number of minutes spent on each of the 32 activities evaluated in this study was startling low. When the need for care was additionally high (Figure 2), there is a need to discuss different possibilities of interpretation of these unexpected results. One explanation may be the low staffing (5 staff in each ward) in both nursing homes. However, the competence of the staff must be considered when deciding the level of staffing. Quality does not entirely depend on the number of staff. Anyhow, a very competent staff can not secure the quality of care without a lower level of staffing in relation to the number of care recipients. Probably there is a relation between the mean number of minutes in each care activity per recipient and the number of available staff on the ward. It is reasonable to raise the question, is the care was sufficient ?. Schnelle et al. [47] ascertained that the majority of nursing homes suffer from significant problems with quality. Quality problems can include: inadequate assistance with eating; little verbal interaction during mealtimes; inadequate assistance with toilet visits and turning of residents; many residents left in bed most of day; little assistance with walking; untreated pain; untreated depression. Similar indications of the existence of problems with the quality of care are also seen in this study. Assistance with mealtimes (nutrition) was one activity that was only allocated 9 minutes during the course of the day. One must therefore strongly question whether this is a sufficient amount of time to ensure that care recipients receive the nutrition they need. Other studies [29, 48] also show that when the time to sit down and help individual patients is insufficient, assistance with mealtimes is an activity often neglected, which may result in malnutrition. For older people, meals are often an important part of the day. Sufficient time must be reserved so that their need for social interaction can be met. Westergren et al. [48] have ascertained that half of all individuals residing at a care home need assistance in order to eat at mealtimes. Consequently, for individual nursing homes, breakfast, lunch, and dinner constitute a peak in work intensity, moments when many hands are needed in order to help residents eat.

The indirect time which indirectly benefits individual care recipients is the time spent by personnel on a ward ensuring that everything runs smoothly and in a satisfactory manner. Cleaning, the preparation of equipment, management of supplies, laundry, food, and movement between various activities are part of the routine work which characterizes daily life. The cleaning of a care recipient's room does not occur together with the care recipient. Instead, care recipients are moved to a common area within the building while the personnel clean so that the personnel can perform this task as quickly as possible. Development has occurred in a direction which makes one question more and more the relevance of asking care personnel to perform work tasks which are not directly related to care work, especially since such tasks are not being used to train and/or stimulate care recipients. 

At the units studied here, care personnel did not participate in the social activities organized for the care recipients in accordance with a prepared weekly schedule at the nursing home. The personnel moved the care recipients that wished to participate into the common area but went back to their unit in order to continue cleaning and/or prepare for lunch or dinner. The care recipients who did not wish to participate in the collective activities often sat alone in their rooms. This meant that many care recipients were alone for a large portion of the day. Social group activities and rehabilitation comprised a total of 13 minutes per day in direct care time, which implies that needs such as exercise and stimulation or time spent outdoors were often limited to a minimum. This has been strongly criticized, mainly by relatives and/or people close to the care recipients. The residents themselves have also expressed that it is important to be able to participate in social activities at some point during the day and to, above all, be able to spend time outdoors. 

A limitation of the study is that the performance of the care has not been assessed.

## 8. Conclusion

The study results show that the care time the care recipients received was limited, and it is reasonable to question whether they received good quality care. Further research is needed before it can be concluded whether the results of this study can be generalized. Also, this study is somewhat weakened in that the description of nursing intensity should be supplemented with components encompassing personnel's proficiency and competence as well as a description of work environment. While analysis of the measurements in this study has shown that the reliability and validity of the TiC-*t *has been satisfactory, further research is nonetheless needed.

##  Competing Interests

The authors declare that they have no competing interests.

##  Authors' Contributions

K. B. E. Thorsell was responsible for and involved in all aspects of the completion of the manuscript. Furthermore, K. B. E. Thorsell was singly responsible for data collection and contacts with the municipal nursing staff. She is a PhD student at Lund University. K. B. E. Thorsell, B. M. Nordström, B. V. Sivberg, and L. Fagerström have together worked on the statistical analysis, instrument development, and interpretation of results. As assistant supervisor, B. M. Nordström contributed to the development and writing of the manuscript. As main supervisor, B. V. Sivberg was responsible for the design of the study and has also participated in all aspects of the study and work with the manuscript. All of the authors have critically reviewed and approved the final manuscript.

## Figures and Tables

**Table:** (a) Direct time in mean minutes per activity from 6.45 AM to 9.15 PM Nursing home 1.

	Period 1				Period 2			
Activity	Number of measurement activities	Mean	Sd	Range	Number of measurement activity	Mean	Sd	Range
Shower/bath	9	18	19	1–55	9	19	17	1–57
Injection	7	2	1	1–4	1	1	—	1–1
KAD	3	5	1	4–6	3	2	1	1–3
Drug	241	3	3	1–36	208	3	3	1–15
Nutrition	155	9	10	1–56	85	10	11	1–60
Hygiene	267	6	5	1–28	193	6	5	1–25
Cognitive Care	49	3	3	1–12	24	3	3	1–10
Rehabilitation	4	7	2	1– 8	2	5	1	1– 6
Social activity	37	6	8	1–35	21	10	12	1–57
Wound	11	5	3	1–11	1	1	—	1– 1
Observation	169	2	2	1–15	153	2	2	1–21
Visits to toilet	244	4	3	1–23	161	4	4	1–33
Transport	252	2	2	1–20	185	3	6	1–73
Other	10	8	4	1–15	7	2	1	1– 8

Total	1458	6	5	1–56	1053	5	5	1–73

*Injections refer to administration of insulin by nursing assistants on delegation.

**Table:** (b) Indirect time in mean minutes per activity from 6.45 AM to 9.15 PM Nursing home 1.

Activity	Number of measurement activities	Mean	Sd	Range	Number of measurement activity	Mean	Sd	Range
Documentation	47	7	6	1–27	24	7	6	1–18
Store	9	7	11	1–36	15	4	5	1–15
Walking time	1394	2	2	1– 38	913	2	2	1–46
Communication	175	4	11	1–123	78	4	5	1–33
Preparing food	252	6	5	1–37	128	8	10	1–59
Report	62	7	5	1–25	59	9	7	1–34
Social activities	31	7	9	1–35	34	11	11	1–43
Cleaning	42	5	4	1–19	17	8	8	1–27
Phone	45	4	15	1–99	24	3	3	1–15
Transport	47	3	3	1–14	44	4	4	1–15
Washing	30	3	4	1–17	6	2	2	1–5
Waiting time	47	4	9	1–50	36	3	2	1– 8
Other	17	8	11	1–36	1	1	—	1–1

Total	2198	5	7	1–123	1379	5	5	1–59

**Table:** (c) Work-place-related time in mean minutes per activity from 6.45 AM to 9.15 PM Nursing home 1.

Activity	Number of measurement activities	Mean	Sd	Range	Number of measurement activity	Mean	Sd	Range
Control	6	4	2	1–8	1	4	—	4–4
equipment								
Personnel	39	3	2	1–8	24	6	16	1– 82
time								
Conference	—	—	—	—	2	11	8	5–16
Phone	23	5	11	1–52	13	2	2	1– 6
Cleaning	25	5	5	1–22	16	13	10	1–40

Total	93	3	4	1–52	56	7	7	1–82

**Table:** (a) Direct time in mean minutes per activity from 6.45 AM to 9.15 PM Nursing home 2.

	Period 1				Period 2			
Activity	Number of measurement activities	Mean	Sd	Range	Number of measurement activity	Mean	Sd	Range
Shower/bath	24	20	13	1–43	15	23	15	1–63
Injection	7	3	2	1– 6	13	4	3	1–10
KAD	5	4	5	1–12	2	2	1	1–2
Drug	175	3	4	1–35	151	3	3	1–18
Nutrition	87	11	10	1–50	62	13	15	1–73
Hygiene	199	7	6	1–26	130	7	5	1–27
Cognitive Care	73	6	9	1–63	25	6	9	1–47
Rehabilitation	5	12	15	1–34	4	11	12	1–40
Social activity	8	8	9	1–27	8	11	14	1–45
Wound	8	5	3	1–10	6	16	21	1–52
Observation	81	2	2	1–11	60	2	3	1–13
Visits to toilet	85	5	4	1–24	75	4	3	1–12
Transport	18	2	3	1–28	39	3	4	1–30
Other	17	5	6	1–24	18	4	8	1–73

Total	792	7	6	1– 63	608	8	8	1–73

*Injections refer to administration of insulin by nursing assistants on delegation.

**Table:** (b) Indirect time in mean minutes per activity from 6.45 AM to 9.15 PM Nursing home 2.

Activity	Number of measurement activities	Mean	Sd	Range	Number of measurement activity	Mean	Sd	Range
Documentation	14	7	4	2–16	15	10	5	1–23
Store	23	5	6	1–26	15	6	5	1–14
Walking time	942	2	2	1–21	775	2	4	1–51
Communication	152	5	6	1–49	133	5	5	1–33
Preparing food	151	7	9	1–91	94	7	6	1–26
Report	54	10	8	1– 37	46	12	10	2–52
Social activities	17	7	8	1–29	17	11	20	1–64
Cleaning	37	9	11	1–54	5	16	9	8–27
Phone	31	3	6	1–33	16	3	2	1–7
Transport	18	2	1	1–28	39	3	2	1–11
Washing	41	3	4	1–15	20	4	5	1–17
Waiting time	73	6	7	1–32	49	5	6	1–24
Other	3	4	3	2–7	4	3	2	1–5

Total	1556	5	6	1–91	1228	7	6	1–64

**Table:** (c) Work- place related time in mean minutes per activity from 6.45 AM to 9.15 PM Nursing home 2.

Activity	Number of measurement activities	Mean	Sd	Range	Number of measurement activity	Mean	Sd	Range
Control	1	1	—	1–1	1	3	—	3–3
equipment								
Personnel	58	8	12	1–48	29	4	3	1–13
time								
Conference	8	90	74	1–148	—	—	—	—
Phone	8	3	3	1– 8	12	4	6	1–10
Cleaning	5	5	5	1–15	3	13	13	4–28

Total	80	21	19	1–148	45	5	4	1–28

**Table 3:** Time in mean minutes/home between 6.45 AM to 9.15 P.M.

	Direct time		Indirect time		Work-place related time	
Nursing home	Period 1	Period 2	Period 1	Period 2	Period 1	Period 2
1	80 (48%)	71 (21%)	67 (41%)	65 (38%)	17 (11%)	36 (21%)
2	93 (35%)	109 (50%)	66 (25%)	84 (39%)	107(40%)*	24 (11%)

Total	173 (40%)	180 (46%)	133 (31%)	149 (38%)	124 (29%)	60 (16%)

Note: *All staff was involved in personnel conference.

**Table 4:** Level of care needs and time spent fulfilling them in mean minutes per day in each unit during periods 1 and 2.

Unit/period	Needs Level	Number of recipients	Mean time (minutes)	Range	Median	Standard devation	Difference in time between needs levels
	1	3	11	4–19	9	8	Kruskal-Wallis
	2	16	44	2–141	17	48	*X* ^2^ = 21, d.f. = 4
1/1	3	14	52	8–119	48	39	*P* < .0003
	4	20	78	16–213	81	47	
	5	26	112	10–253	100	70	

	1	3	18	5–40	10	19	Kruskal-Wallis
	2	16	40	3–106	41	27	*X* ^2^ = 23. d.f. = 4
1/2	3	14	49	10– 97	46	29	*P* ≤ .0001
	4	20	74	12–173	72	40	
	5	26	96	13–204	90	52	

	1	6	26	8–72	14	25	Kruskal-Wallis
	2	16	46	7–110	35	33	*X* ^2^ = 32. d.f. = 4
2/1	3	14	62	15–154	56	35	*P* ≤ .0001
	4	15	80	39–128	72	31	
	5	29	148	19–378*	129	84	

	1	6	16	4–50	8	18	Kruskal-Wallis
	2	16	34	2–112	28	33	*X* ^2^ = 40. d.f. = 4
2/2	3	14	43	10–130	32	37	*P* ≤ , .0001
	4	15	63	7–144	51	39	
	5	29	105	34–291	90	70	

Note: ^  ∗^The  high number of minutes is due to the fact that at the time of measurement two care recipients required significant amounts of care.
